# Audit of Conservative Management of Acute Ureteric Stones and Factors Influencing Treatment Outcome in the Emergency Department

**DOI:** 10.7759/cureus.117417

**Published:** 2026-10-04

**Authors:** Ifeanyichukwu E Ihedoro, Ebuka Egwuatu, Lesedi Leepetswe, Ahmed Abroaf, Andrei Dontu, Kathie Wong, Jaswant Mom, Syed Rizvi, Dumitru Butu

**Affiliations:** 1 Urology, Eleos Specialist Hospital, Umuahia, NGA; 2 Urology, North Cumbria Integrated Care NHS Foundation Trust, Carlisle, GBR; 3 Urology, Cumberland Infirmary, Carlisle, GBR; 4 Surgery, Cumberland Infirmary, Carlisle, GBR; 5 Urology, West Cumberland Hospital, Carlisle, GBR

**Keywords:** conservative, nsaids, stone, tamsulosin, ureteric stone

## Abstract

Background: Ureteric stones are a common cause of presentation to the emergency department. Management options include conservative therapy, urinary diversion (ureteric stent /nephrostomy), ureteroscopy, or extracorporeal shockwave lithotripsy. Conservative management is known to have a high success rate in appropriately selected patients. This audit evaluates compliance with evidence-based standards in acute ureteric stones management and identifies factors influencing successful conservative treatment.

Methods: This retrospective audit reviewed 143 patients who presented with renal colic to the Emergency Department between January and December 2025 and were offered conservative management. Data collected included patient demographics, laboratory findings, CT parameters, administration of non-steroidal anti-inflammatory drugs (NSAIDS) and tamsulosin, re-presentation rates and treatment outcomes. The audit benchmarked against NICE guidelines. Data analysis was performed using descriptive statistics and appropriate statistical tests (t-test, Fisher’s exact test and Mann-Whitney U test) with significance set at p < 0.05.

Results: A total of 143 patients met the inclusion criteria. The average age of patients was 48 years. This comprised 101 males (70.63%) and 42 females (29.37%). NSAIDS were administered to 103 patients (72%) at presentation, while 49 patients (34.3%) with distal ureteric stones received tamsulosin. The success rate for the conservative therapy was 93.7%. Seventeen patients (11.9%) re-presented to the emergency department with colic. Among all the variables analyzed (C-reactive protein, white blood cell (WBC), creatinine, perinephric stranding, stone position), stone size was the only variable that showed a significant association with the outcome of conservative treatment (p< 0.05).

Conclusion: Our audit findings revealed that NSAIDS and tamsulosin were sub-optimally administered in eligible patients; however, there was a very high rate of successful conservative treatment. Stone size was the only variable that showed significant association with successful conservative treatment.

## Introduction

The management of acute ureteric stones represents a frequent and challenging scenario in urological emergency departments worldwide [1]. While surgical interventions such as shock wave lithotripsy or ureteroscopy offer definitive stone clearance, conservative management primarily through active surveillance and medical expulsive therapy remains the preferred first-line approach for most small, uncomplicated calculi [1,2]. This strategy capitalizes on the natural history of small ureteric stones, which frequently pass spontaneously, thereby sparing patients from potential surgical morbidities, anesthesia risks, and substantial healthcare costs [3]. However, the success of a conservative approach is highly variable, and prolonged, unsuccessful observation can lead to severe complications, including persistent pain, urinary tract infections, and irreversible renal impairment [4]. Consequently, identifying the specific factors influencing treatment outcomes, such as stone size, anatomical location, degree of hydronephrosis, and patient-specific biomarkers, is critical to refining clinical decision-making [3,5]. Hence, this audit aims to evaluate compliance with evidence-based standards in acute ureteric stone management and identify factors influencing successful conservative treatment.

## Materials and methods

This is a retrospective audit of patients with renal colic from ureteric stones at North Cumbria Integrated Care NHS Foundation Trust. The data for this audit was collected from 1st January to 31st December 2025.

One hundred and forty-three (143) patients were included in the study. All adult patients who were offered conservative management for ureteric stones were included in the study, while those who had emergency urinary diversion (ureteric stent or nephrostomy) and patients in the paediatric age group were excluded from the study.

Approval was obtained from the trust-wide Clinical Audit Triage Group (2486). The data was collected from the attendance notes of the patients and transferred to an Excel sheet (Microsoft, Redmond, WA, USA) proforma. Data collected included patient demographics, laboratory findings (serum creatinine, white blood cell count (WBC), C-reactive protein (CRP)), CT parameters (stone size, stone position, perinephric stranding), administration of non-steroidal anti-inflammatory drugs (NSAIDS) and tamsulosin, re-presentation rates and treatment outcome.

The audit was benchmarked against the National Institute for Health and Care Excellence (NICE) guidelines. The NICE guidelines recommend that patients on conservative therapy for ureteric stones should receive NSAIDS as the first-line analgesia, unless contraindicated. It also recommends that tamsulosin should be prescribed to patients who have stones of less than 10mm in the distal ureter [6].

Continuous variables were reported as means with corresponding standard deviations, while categorical variables were summarized as frequencies and proportions. Data analysis was performed using descriptive statistics and appropriate statistical tests (t-test, Fisher’s exact test and Mann-Whitney U test) with significance set at p < 0.05.

## Results

A total of 143 patients were included in this audit. The mean age of the participants was 47.99 +/- 15.6 years, as shown in Figure 1. One hundred and one patients were males (70.63%), while 42 were females (29.37%), as shown in Figure 2. Eighty patients (55.94%) had ureteric stones less than 5mm, while 61 patients (42.66%) had stones measuring 5-10mm. Two patients with stone size of more than 10mm opted for conservative management, as demonstrated in Figure 3. One hundred and fifteen patients (80.42%) had distal ureteric stones, while 20 patients (13.99%) had proximal ureteric stones, as shown in Figure 4. Seventeen patients (11.9%) re-presented to the emergency department following commencement of conservative treatment. Eight of these patients went on to have successful conservative treatment, while nine patients had emergency intervention (eight patients had emergency ureteric stent insertion, while one had ureteroscopy). One hundred and thirty-four patients (93.7%) had successful conservative treatment. Forty-nine patients (34.3%) with distal ureteric stone had tamsulosin prescription at presentation, while 103 (72%) were offered NSAIDS during the acute episode (Table 1). Stone size showed a significant association with the outcome of conservative treatment, while CRP, WBC, creatinine, perinephric stranding, and stone position did not show any significant association (p< 0.05), as shown in Table 2.

**Figure 1:**
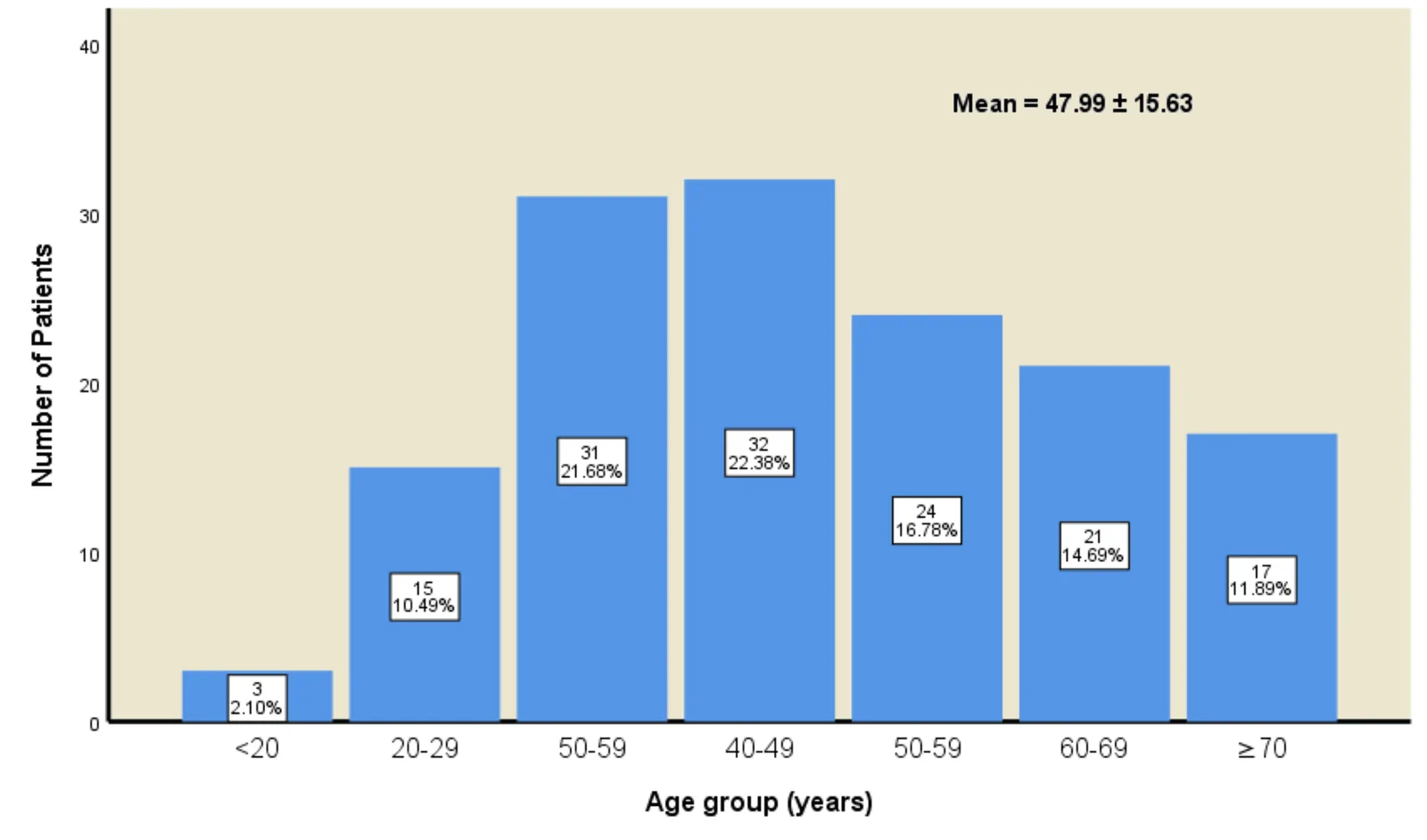
Age Distribution of the Patients

**Figure 2:**
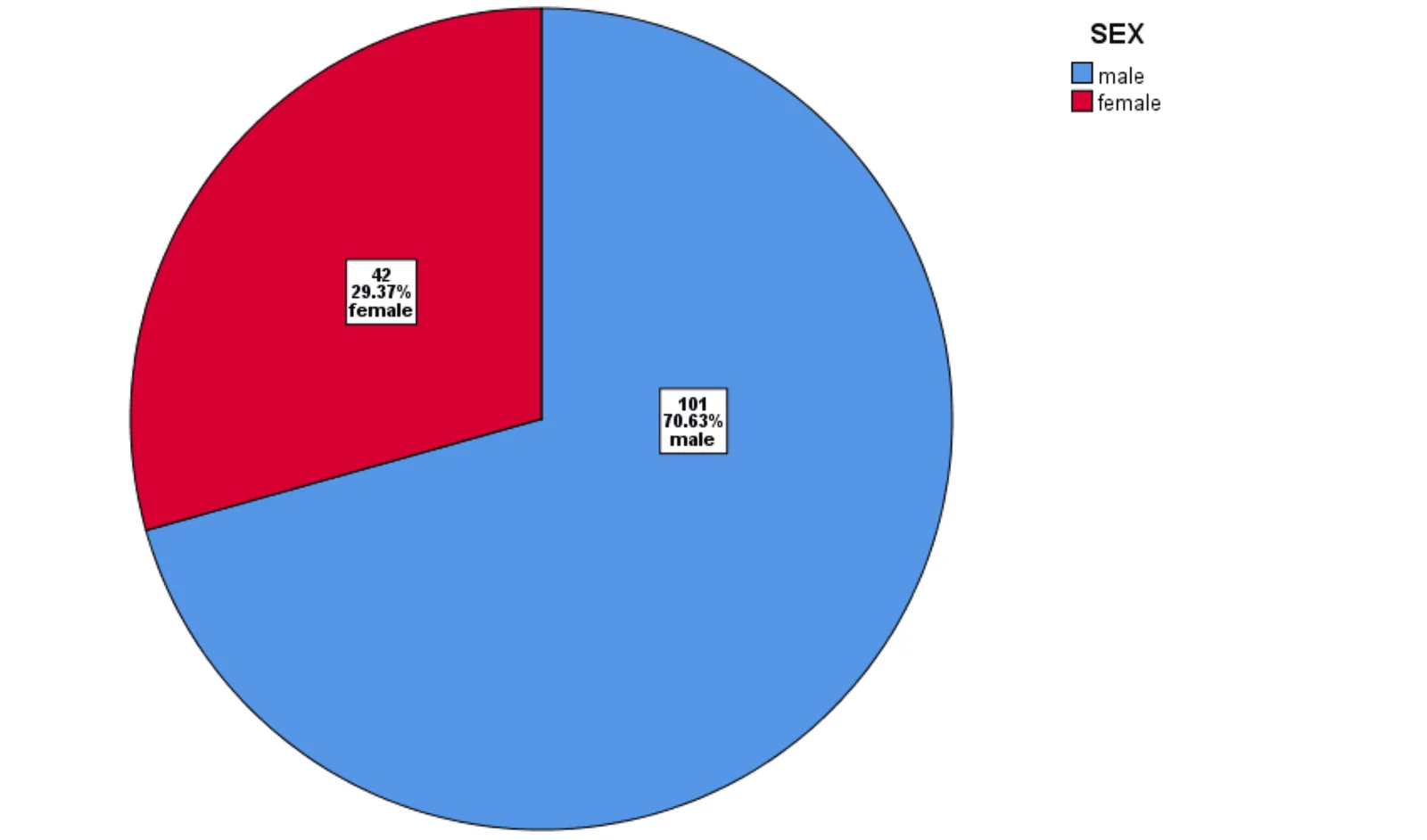
Sex Distribution of the Patients

**Figure 3:**
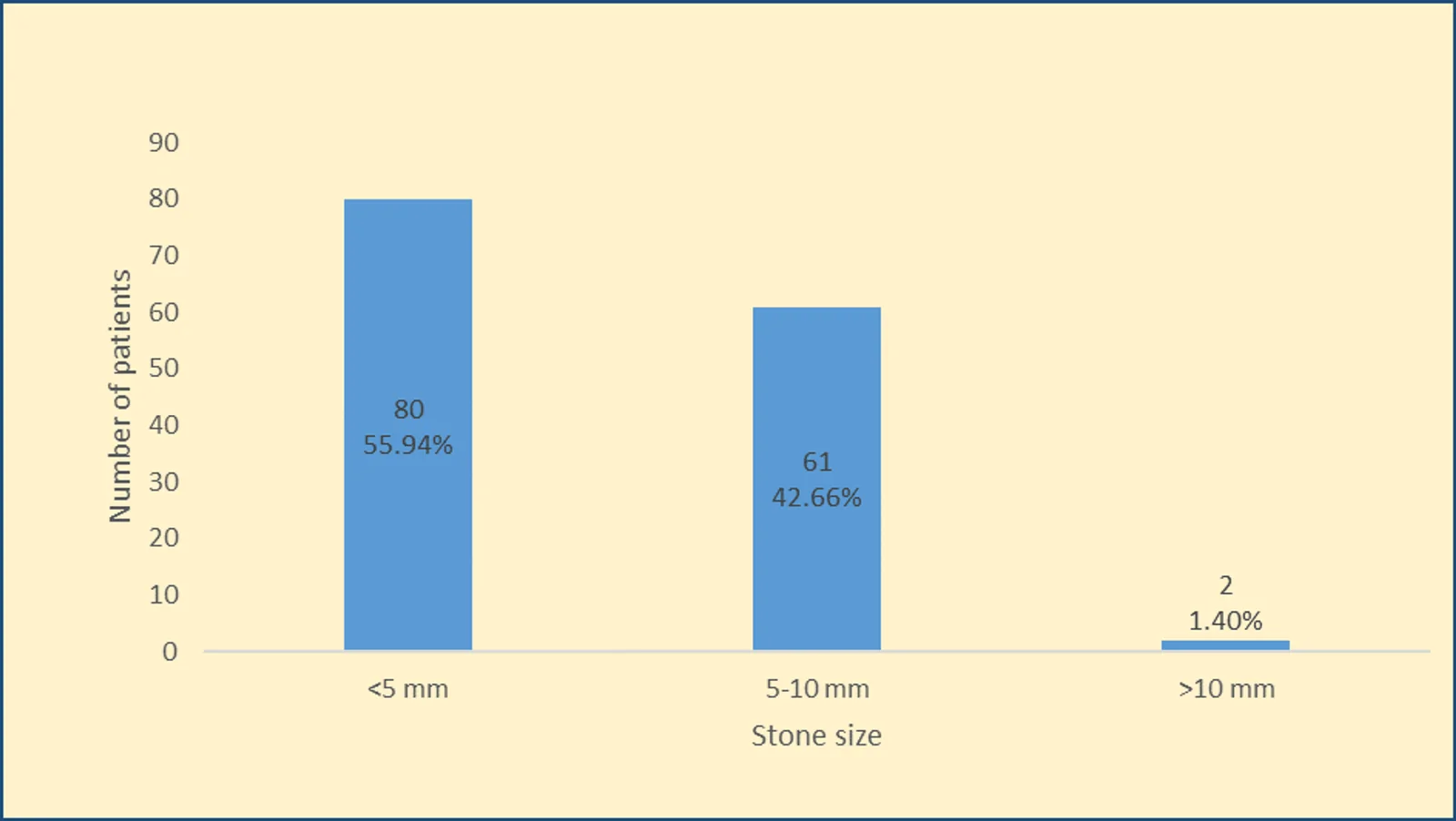
Size of the ureteric stones

**Figure 4:**
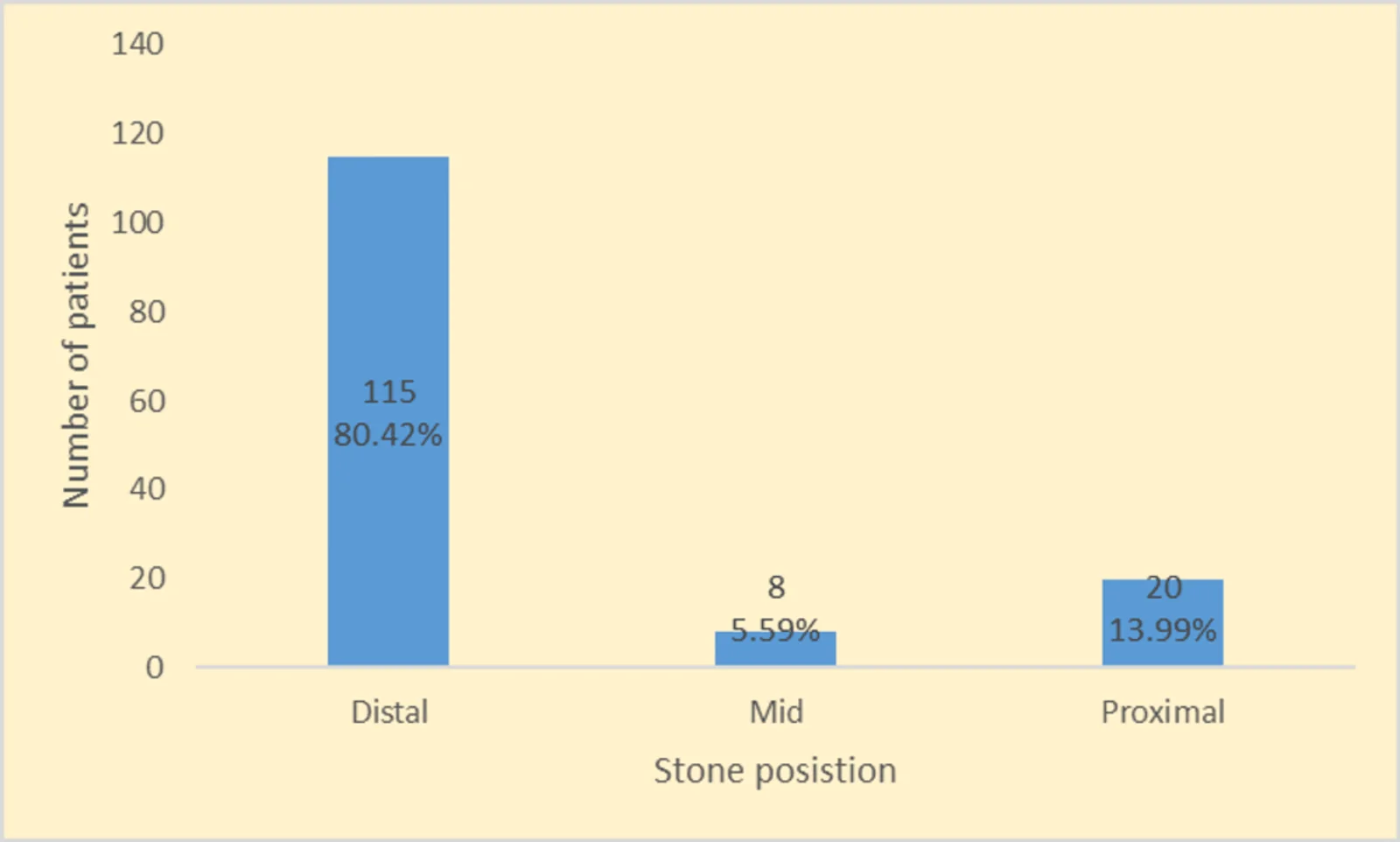
Position of Stones in the Ureter

**Table 1:** Clinical Parameters and Treatment Outcome Among the Patients NSAID: non-steroidal anti-inflammatory drug

Variables	Frequency, n = 143	Percent (%)
Re-presentation		
Yes	17	11.9
No	126	88.1
Success of treatment		
Successful	134	93.7
Failed conservative	9	6.3
Tamsulosin		
Yes	49	34.3
No	94	65.7
NSAID given		
Yes	103	72.0
No	40	28.0

**Table 2:** Relationship between Clinical Parameters and Treatment Outcome Among the Patients *significant, f: Fisher’s exact test, t: t-test, u: Mann-Whitney U test.

Variables	Conservative Therapy		Fisher’s exact test/t-test/Mann-Whitney U test	P value
	Successful, n (%)	Failed, n (%)		
Stone size (mm)				
<5	79(98.8)	1(1.3)	16.547^f^	<0.001*
5-10	55(90.2)	6(9.8)		
>10	0(0.0)	2(100.0)		
C-Reactive Protein				
<2	67(93.1)	5(6.9)	0.054^f^	0.816
≥2	63(94.0)	4(6.0)		
Perinephric Stranding				
Yes	76(92.7)	6(7.3)	0.341^f^	0.733
No	58(95.1)	3(4.9)		
Stone’s position				
Distal	110(95.7)	5(4.3)	6.997^f^	0.051
Mid	8(100.0)	0(0.0)		
Proximal	16(80)	4(20)		
Tamsulosin (Distal stone only)				
Yes	43(97.7)	1(2.3)	0.738^f^	0.648
No	67(94.4)	4(5.6)		
White Blood Cell (mean±SD)	11.2±3.2	10.1±3.5	0.972^t^	0.333
Creatinine (Median(IQR))	91.0(78.8- 109.0)	93.0(75.0-119.5)	549.000^ u^	0.653

## Discussion

The demographics of the patients attending the clinic under this audit align closely with the established global epidemiological profile of urolithiasis, which characteristically peaks between the third and fifth decades of life and shows a higher prevalence in men [1]. In the acute setting, adequate analgesia is paramount; NSAIDs were administered to most of the patients at presentation. This high rate of utilization reflects international guidelines, such as the NICE guidelines and the European Association of Urology (EAU) guidelines, which advocate for NSAIDs as the first-line analgesic modality due to their efficacy in reducing both ureteral smooth muscle spasms and prostaglandin-mediated renal pelvic pressure [2,6]. Furthermore, medical expulsive therapy (MET) using the alpha-1 adrenoreceptor antagonist tamsulosin was prescribed to only about one-third of patients presenting with distal ureteric stones. Tamsulosin selectively targets alpha-1A and alpha-1D receptors, which are highly concentrated in the distal third of the ureter [1,2]. By inhibiting basal smooth muscle tone and reducing peristaltic amplitude, tamsulosin lowers intraluminal pressure gradients to facilitate stone expulsion, a selective practice supported by contemporary clinical trials and systematic reviews for lower ureteric calculi [7,8]. We considered tamsulosin administration for patients with distal ureteric stones, who had no features of sepsis, persistent renal colic despite adequate analgesia or significant renal compromise. 

The overall success rate for conservative therapy among patients in our audit was remarkably high. Yallappa S et al. carried out a systematic review involving 6,600 patients who had conservative management of ureteric stones. They reported that 64% of the patients had successful conservative treatment [9]. Conservative therapy, paired with appropriate medical treatment, remains an exceptionally viable, effective, and safe first-line strategy for uncomplicated ureteric calculi, effectively sparing the vast majority of patients from the potential morbidities and substantial healthcare costs associated with early, invasive surgical interventions like ureteroscopy or shockwave lithotripsy [7,10]. Ideally, it should be offered to patients who have not presented with sepsis, persistent renal colic despite adequate analgesia, or renal decline [9]. Nonetheless, conservative management is not devoid of clinical challenges; a subset of patients re-presented to the emergency department experiencing recurrent renal colic. This highlights the volatile natural history of migrating calculi and underscores the vital importance of strict outpatient follow-up protocols, clear patient counselling regarding warning signs, and vigilant monitoring to prevent silent, long-term renal deterioration [7,11]. The British Association of Urological Surgeons recommends that active treatment be offered to patients who have not had evidence of successful conservative treatment after two to three weeks [12]. Most UK-based urological surgeons advocate for follow-up imaging to confirm successful stone passage [13].

When evaluating potential clinical, laboratory, and radiological markers of spontaneous passage, stone size emerged as the only variable demonstrating a statistically significant association with conservative treatment outcomes. Interestingly, inflammatory and biochemical biomarkers, including CRP, WBC count, and serum creatinine, were not associated with the treatment outcome in this audit. This indicates that while systemic inflammatory markers are invaluable for ruling out concurrent septic urological emergencies which require urgent surgical decompression, they do not reliably reflect the mechanical likelihood of a sterile calculus navigating the ureteral lumen [2,7]. Similarly, radiological features such as the stone's anatomical position and the presence of perinephric stranding were not significantly associated with treatment success. While some historical literature emphasizes a higher spontaneous clearance rate for distal versus proximal stones [3], our finding that stone size uniquely dictates the therapeutic outcome strongly supports the anatomical reality that the absolute physical dimensions of the calculus represent the primary limiting factor when negotiating the narrowest physiological segments of the ureter [3,8]. Consequently, structural stone size must remain the core metric utilized by clinicians to establish safe observation windows, select candidates for conservative protocols, and determine the optimal timing for secondary surgical intervention [2,11]. Similar studies have however reported that stone size, location, underlying diabetes mellitus, and perinephric fat stranding are statistically significant predictors of successful conservative treatment [14,15].

This study has some limitations. It is a single-centre retrospective audit. Therefore, the findings may not be generalizable to other clinical settings. A prospective multi-institute study will be more suitable. A relatively small cohort of patients had unsuccessful conservative therapy. This may have had a negative impact in the ability of the study to correctly predict significant associations between clinical and laboratory factors and successful conservative treatment.

## Conclusions

Our audit revealed that conservative treatment for management of acute ureteric stones has significantly high success rates, with stone size being the only statistically significant predictor of treatment outcome. However, there is a need to improve compliance with NICE guidelines in the use of NSAIDS as first-line analgesia and prescription of tamsulosin. Increased awareness of national and local guidelines will play an important role in optimising compliance and improving the patients' experience.

## References

[1] Pearle MS, Goldfarb DS, Assimos DG (2014). Medical management of kidney stones: AUA guideline. J Urol.

[2] Skolarikos A, Geraghty R, Somani B (2025). European Association of Urology guidelines on the diagnosis and treatment of urolithiasis. Eur Urol.

[3] Coll DM, Varanelli MJ, Smith RC (2002). Relationship of spontaneous passage of ureteral calculi to stone size and location as revealed by unenhanced helical CT. AJR Am J Roentgenol.

[4] Dassanayake SN, Harrison NL, Geraghty R (2026). Between a stone and a hard place - mortality from conservative management of kidney stone disease: systematic review of the literature from the European Association of Urology Section of Endourology. Asian J Urol.

[5] Sfoungaristos S, Kavouras A, Perimenis P (2012). Predictors for spontaneous stone passage in patients with renal colic secondary to ureteral calculi. Int Urol Nephrol.

[6] (2026). Renal or ureteric colic - acute management. https://cks.nice.org.uk/topics/renal-or-ureteric-colic-acute/background-information/prevalence/.

[7] Shah TT, Gao C, Peters M (2019). Factors associated with spontaneous stone passage in a contemporary cohort of patients presenting with acute ureteric colic: results from the multi-centre cohort study evaluating the role of inflammatory markers in patients presenting with acute ureteric colic (MIMIC) study. BJU Int.

[8] Pickard R, Starr K, MacLennan G (2015). Medical expulsive therapy in adults with ureteric colic: a multicentre, randomised, placebo-controlled trial. Lancet.

[9] Yallappa S, Amer T, Jones P (2018). Natural history of conservatively managed ureteral stones: analysis of 6600 patients. J Endourol.

[10] Subramonian K, Barba HS, Darrad M (2020). Role of conservative management of stones. Turk J Urol.

[11] Preminger GM, Tiselius HG, Assimos DG (2007). 2007 guideline for the management of ureteral calculi. J Urol.

[12] (2026). Kidney stones. https://www.baus.org.uk/patients/conditions/6/kidney_stones/.

[13] Jarral F, Hamdy A, Wrazen J, Mohamed G, Abusanad O (2024). Follow-up survey for conservatively managed ureteric stones. Cureus.

[14] Choi T, Yoo KH, Choi SK (2015). Analysis of factors affecting spontaneous expulsion of ureteral stones that may predict unfavorable outcomes during watchful waiting periods: what is the influence of diabetes mellitus on the ureter?. Korean J Urol.

[15] Ahmed AF, Gabr AH, Emara AA, Ali M, Abdel-Aziz AS, Alshahrani S (2015). Factors predicting the spontaneous passage of a ureteric calculus of ⩽10 mm. Arab J Urol.

